# Accuracy Evaluation Method for Blade Vibration Measurement in Blade Tip Timing Based on Direct Calibration Using Time of Arrival

**DOI:** 10.3390/s25071956

**Published:** 2025-03-21

**Authors:** Qi Zhou, Guangyue Niu, Meiru Liu, Guangrong Teng, Fajie Duan, Fangyi Li, Hao Liu, Fafu Li

**Affiliations:** 1 State Key Laboratory of Precision Measurement Technology and Instruments, Tianjin University, Tianjin 300072, China; zhouqi1023@tju.edu.cn (Q.Z.); fjduan@tju.edu.cn (F.D.); lfy0424@tju.edu.cn (F.L.); liu_hao@tju.edu.cn (H.L.); 1023202041@tju.edu.cn (F.L.); 2 AECC Sichuan Gas Turbine Establishment, Chengdu 610500, China; 18784010242@163.com (M.L.); tenggr2025@163.com (G.T.)

**Keywords:** blade vibration, blade tip timing (BTT), time of arrival (ToA), direct calibration, error analysis, uncertainty analysis

## Abstract

Non-contact blade vibration measurement based on blade tip timing (BTT) is a signature method for health monitoring in large rotating machinery. Time of arrival (ToA), as the fundamental data in BTT, directly impacts the accuracy of subsequent vibration parameter identification, thereby affecting the effectiveness of real-time condition monitoring and fault detection. However, no direct calibration method currently exists for ToA, and BTT errors are typically assessed through indirect or relative measurements, resulting in imprecise accuracy evaluations. To address this gap, this paper proposes a method for evaluating BTT measurement accuracy through direct calibration of ToA. A ToA direct calibration model is developed, which equivalently transforms the ToA variation caused by blade vibration into the circumferential angle difference between the BTT sensor and the rotating blade disk. The associated errors are systematically analyzed, and the BTT measurement accuracy is assessed using the directly calibrated ToA. Additionally, a BTT accuracy evaluation device was developed to facilitate this assessment. The uncertainty of the device was evaluated using the Monte Carlo method, accounting for both systematic and random factors. At 0.5° and 1000 rpm, the device yielded an estimated ToA value of 83.3055 μs, with the standard uncertainty of 8.824 × 10^−3^ μs and the 95% confidence interval of [83.2881, 83.3233] μs. The accuracy evaluation tests performed with the developed device simulated various vibration displacement and rotational speed conditions to validate the optical fiber BTT measurement system. The results showed that the system achieved a relative accuracy better than 0.8% and a repeatability accuracy exceeding 0.5%. The proposed BTT accuracy evaluation method and device have been validated for assessing both the accuracy and stability of the BTT measurement system, providing a reliable and precise approach for its evaluation.

## 1. Introduction

High-speed rotating blades are core components of large rotating machinery, such as aero-engines [1,2], gas turbines [3,4], and steam turbines [5]. Their operational status directly impacts the safety and efficiency of the equipment. Blade vibration directly reflects the operating status of the blade. Blade tip timing (BTT) method was first proposed by I. E. Sabuloski in the 1960s [6]. It has the advantages of non-contact, low intervention, and real-time measurement of all blades. It has been verified by Peter Russhard, chief engineer of Rolls Royce in the UK and other industry experts [7], and has been included in the aero-engine test outline by European and American countries [8]. The method is widely used in blade vibration measurement [9,10,11] and fault diagnosis [12,13,14]. The basic principle of BTT method is to use BTT sensors to detect the time difference when a blade reaches the BTT sensor, caused by the vibration of the blade, which allows for precise measurement of blade vibrations. Therefore, time of arrival (ToA) is the basic data of BTT method, and its measurement accuracy directly determines the accuracy of the subsequent vibration parameter identification.

At present, the research on the BTT method is more focused on the blade vibration parameters identification [15,16,17,18]. In the study of ToA extraction, there are frontier moment method [19], peak moment method [20], second derivative moment method [21], AGC circuit compensation method [22], double-edge moment method [23,24], and signal centroid extraction method [25], etc. These methods are typically evaluated through comparative studies against existing or unimproved approaches to demonstrate the advancements of the newly proposed methods. However, such comparative evaluations can only reveal the relative optimization degree of measurement accuracy, making it challenging to establish an accurate and reliable standalone method. This limitation arises because, to rigorously test the accuracy of a proposed method, the ideal approach is to compare the ToA results with measurements obtained using an independent reference method. Theoretically, this independent method must exhibit higher accuracy and reliability than the ToA-based measurements. Unfortunately, within the framework of the BTT method itself, no such independent reference method currently exists.

Among the existing methods for evaluating the measurement accuracy of BTT, the most widely applied and mature approach is the use of strain gauges [26,27,28]. However, due to the inherent limitations of the strain gauge principle, this method is primarily reliable for evaluating the accuracy of vibration frequency. In addition, the measurement accuracy is verified by vibration displacement, but the reliability is lacking. J. Gallego-Garrido [29] proposed a blade visualization method that employs a high-speed camera to record vibration displacement. While this approach offers a novel means of visualizing blade vibrations, it is constrained by the camera’s exposure time when the blade’s rotational speed is excessively high, which adversely affects image quality. Notably, the accuracy of this method remains unverified, and its performance under high-speed conditions has not been rigorously evaluated. D. Di Maio [30] suggested the synchronous application of Scanning Laser Doppler Vibrometers and the BTT measurement system. However, their experimental results showed significant discrepancies, highlighting the need for further investigation to reconcile these differences. In addition, Yiming Meng [31] and Pengfei Chai [32] proposed the use of laser displacement sensors to obtain accurate measurements of blade tip displacement. However, their method relies on finite element analysis to establish the proportional relationship between measured displacement and actual blade vibration, which introduces additional computational complexity and potential sources of error.

In summary, there is a lack of systematic calibration methods for ToA in BTT measurements. The evaluation of BTT errors largely relies on indirect or relative measurement approaches, and comprehensive error analysis and uncertainty analysis have not been systematically conducted. This limitation poses significant challenges in verifying measurement accuracy, thereby undermining the reliability of BTT error evaluation and its application in high-precision scenarios. Addressing these issues would provide a foundation for more accurate and reliable BTT measurement systems.

In this paper, a direct calibration method for ToA based on the circumferential angle difference is proposed, leveraging BTT method and the principle of ToA formation. In this approach, the ToA variation caused by blade vibration is equivalent to the circumferential angle difference in the BTT sensor relative to the rotating blade disk. A direct calibration model for ToA is constructed, and the errors introduced by the circumferential angle difference are analyzed. Using the ToA obtained through direct calibration, the accuracy of BTT measurements is evaluated. To support this analysis, a BTT accuracy evaluation device is developed. Considering the influence of system and environmental factors, an uncertainty analysis model is established, and the uncertainty of the device is quantified using the Monte Carlo method. The developed device is then applied to evaluate the accuracy of an optical fiber-based BTT measurement system under simulated conditions of varying vibration displacements and rotational speeds.

The structure of this paper is as follows: Section 2 introduces the evaluation method for BTT measurement accuracy, including the construction of the ToA direct calibration model and the analysis of its associated errors. Section 3 presents the design of the BTT accuracy evaluation device and an analysis of its measurement uncertainty. In Section 4, the developed device is utilized to evaluate the accuracy of an optical fiber-based BTT measurement system. Finally, Section 5 summarizes the findings and outlines future research directions.

## 2. Accuracy Evaluation Method for Blade Tip Timing

### 2.1. Direct Calibration Method of Time of Arrival Based on Circumferential Angle Difference

**Figure 1:**
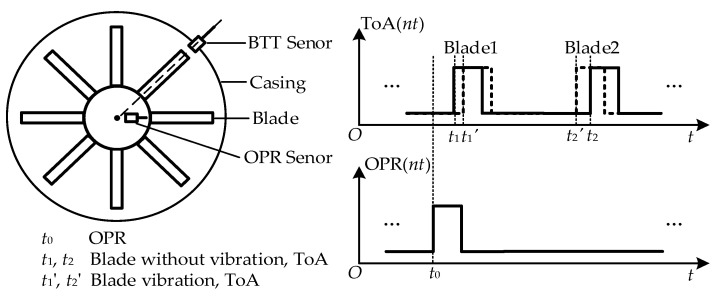
BTT measurement principle.

The principle of BTT measurement is illustrated in Figure 1. The vibration displacement of blade #*j* (*j* = 1, 2, …, *J*) measured by BTT sensor *i* (*i* = 1, 2, …, *I*) is denoted as *y_ij_*. When blade *j* is stationary (i.e., without vibration), the ToA at sensor *i* is *t′_ij_*. When blade *j* vibrates, the ToA at sensor *i* becomes *t_ij_*. The ToA value is related to the rotational speed of the blade and its vibration characteristics. The ToA difference Δ*t_ij_* (i.e., *t_ij_* − *t′_ij_*) is induced by the blade vibration and encodes the blade vibration information. By utilizing the ToA difference Δ*t_ij_*, combined with the known rotational speed, the blade vibration displacement *y_ij_* is calculated as follows:

(1) $$y_{ij}=r\omega \cdot (t_{ij}-t_{ij}^{\prime })=r\omega \cdot \Delta t_{ij}$$

where *r* is rotor radius, and *ω* is rotor angular velocity. When using the BTT method for calculations, it is essential that the rotor angular velocity ω remains known and constant throughout a single computational process. Therefore, all quantities related to angular velocity, both here and in subsequent analyses, should be considered predetermined and constant.

The error of blade vibration displacement *y_ij_* is calculated as

(2) $$\frac{\Delta y_{ij}}{y_{ij}}=\frac{\Delta r}{r}+\frac{\Delta \omega }{\omega }+\frac{\Delta (\Delta t_{ij})}{\Delta t_{ij}}$$

where the first two items are related to the measured blade disk, and the third item is related to the ToA measurement accuracy of the BTT measurement system.

**Figure 2:**
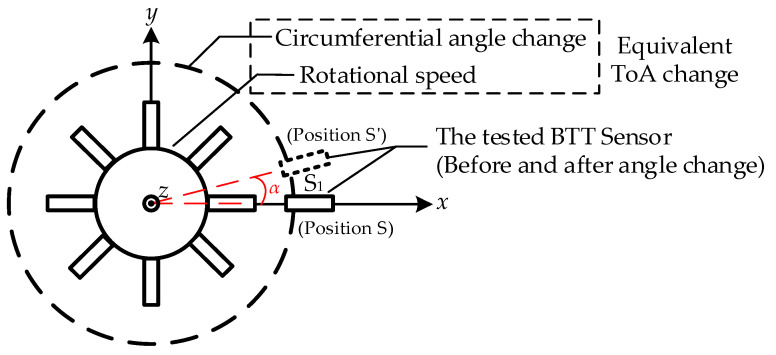
ToA direct calibration model based on circumferential angle change.

Changes in ToA caused by blade vibration, whether leading or lagging, form the fundamental basis of the BTT measurement principle. This study is based on the BTT principle and the formation mechanism of ToA. It proposes a direct calibration method for ToA, where changes in ToA induced by blade vibration are equivalently transformed into a combination of the circumferential angle variation in the BTT sensor and the rotational speed. From this, a ToA direct calibration model based on circumferential angle differences is established, as depicted in Figure 2.

The change in circumferential angle causes the tested BTT sensor S_1_ to rotate by an angle of *α*. The theoretical value of the ToA before and after the same blade reaches S_1_ is calculated as

(3) $$\Delta t_{j}=\frac{\alpha }{\omega }$$

At this time, the ToA measurement obtained by the BTT sensor is represented as Δ*ToA_j_*. The relationship between the theoretical ToA and the measured ToA can be rep-resented using polynomial fitting, as expressed in

(4) $$\Delta ToA_{j}=a_{n}{\left(\Delta t_{j}\right)}^{n}+a_{n-1}{\left(\Delta t_{j}\right)}^{n-1}+a_{n-2}{\left(\Delta t_{j}\right)}^{n-2}+\ldots +a_{1}(\Delta t_{j})+a_{0}$$

The coefficients *a_n_*, *a_n_*_-1_, *a_n_*_-2_, …, *a*_1,_ and *a*_0_ can be determined through calibration, enabling the direct calibration of the ToA measurement for the BTT sensor.

### 2.2. Blade Tip Timing Accuracy Evaluation Method Based on Time of Arrival

**Figure 3:**
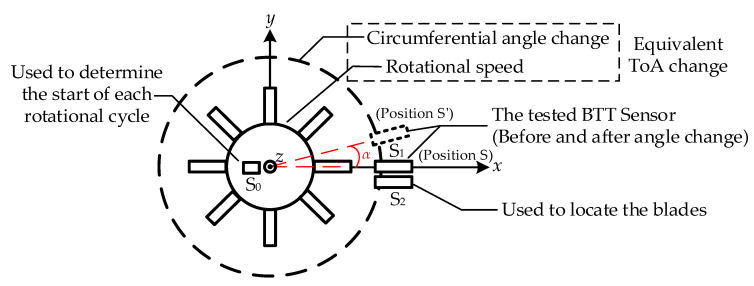
BTT accuracy evaluation model based on ToA.

As the fundamental data in the BTT method, ToA directly reflects the blade vibration characteristics and serves as the foundation for accuracy evaluation. Building on the ToA direct calibration method in Section 2.1, this paper develops a BTT accuracy evaluation approach depicted in Figure 3. By identifying the start of each rotation and applying ToA differential measurements, ToA values are obtained under varying rotation counts and for different blades. The accuracy evaluation is conducted by comparing the theoretical ToA values and the measured values.

In this model, sensor S_0_ is used to determine the start of each rotational cycle, while sensor S_2_ is employed to identify individual blades. The measurement results from sensors S_1_ and S_2_ are processed using a differential method. Specifically, the difference in the ToA values before and after rotation of S_1_ is calculated as

(5) $$f(\Delta S_{1})=f(\Delta {S_{12}}^{\prime })-f(\Delta S_{12})$$

where *f*(Δ*S*_12_) is the ToA difference between the tested BTT sensor S_1_ and sensor S_2_ before the rotation of S_1_, while *f*(Δ*S*_12_′) represents the corresponding ToA difference after rotation. Since sensor S_2_ is fixed, the ToA measurements for the blade passing through S_1_ before and after rotation can be calculated by taking the difference between these two measurements.

The first blade that passes through S_1_ after marking point on the shaft passes S_0_ is defined as the blade #1. The test duration is *T* (s), and the rotational speed is *n_r_* (rpm, where *n_r_ =* 60*ω*/2π), and the effective number of cycles is *K* (where *K* ≤ *n_r_*/60 × *T*). As the blade passes the BTT sensor, it generates a blade-tip sensing signal *q*(*t*), which is sampled and quantized at discrete time intervals *t_k_* to yield *q*(*nt_k_*). Each blade provides a single ToA, forming the BTT signal *ToA*(*nt_k_*). The initial BTT signals from S_1_ and S_2_, denoted as *ToA*_1_(*nt_k_*) and *ToA*_2_(*nt_k_*), can be expressed as *ToA*_1_(*j*,*k*) and *ToA*_2_(*j*,*k*), where *j* represents the blade index, and *k* denotes the rotation cycle index.

(6) ToA1(j,k)=ToA1(ntk)=ToA1,11ToA1,12…ToA1,1KToA1,21   … … ToA1,J1  ToA1,JK

(7) ToA2(j,k)=ToA2(ntk)=ToA2,11ToA2,12…ToA2,1KToA2,21   … … ToA2,J1  ToA2,JK

The initial OPR signal *q*_0_(*nt_k_*) from S_0_ is represented as *T*_0_(*k*) for each rotation cycle. This signal provides the reference time *T*_0_(*k*), which serves as the basis for aligning and analyzing ToA measurements across cycles.

(8) $$T_{0}(k)=q_{0}(nt_{k})=\left[\begin{array}{cccc} T_{01} & T_{02} & \ldots & T_{0K} \end{array}\right]$$

From Equations (6) and (7), the BTT signal difference between S_1_ and S_2_ is derived as

(9) ΔToA12(j,k)=ToA1(j,k)−ToA2(j,k)=ToA1,11-ToA2,11ToA1,12-ToA2,12…ToA1,1K-ToA2,1KToA1,21-ToA2,21   … … ToA1,J1-ToA2,J1  ToA1,JK-ToA2,JK

To mitigate the impact of random errors, a multi-round averaging method is applied to Equation (9), and it is calculated as

(10) $$\Delta ToA_{12}(j)=\bar{\Delta ToA_{12}(j,k)}=\frac{1}{K}\left[\begin{array}{c} \sum_{k=1}^{K}\left(ToA_{1,1k}-ToA_{2,1k}\right)\\ \sum_{k=1}^{K}\left(ToA_{1,2k}-ToA_{2,2k}\right)\\ \ldots \\ \sum_{k=1}^{K}\left(ToA_{1,Jk}-ToA_{2,Jk}\right) \end{array}\right]$$

After adjusting the circumferential angle *α*, the test parameters are updated, with the test duration denoted as *T’* and the rotation speed as *n_r_’*. The effective number of cycles, *K’*, satisfies *K’* ≤ *n_r_’*/60 × *T’*. Under these adjusted parameters, the BTT signal difference between S_1_ and S_2_ is calculated as

(11) $$\Delta To{A_{12}}^{\prime }(j,k)=To{A_{1}}^{\prime }(j,k)-To{A_{2}}^{\prime }(j,k)$$

After averaging, it is calculated as

(12) $$\Delta To{A_{12}}^{\prime }(j)=\bar{\Delta To{A_{12}}^{\prime }(j,k)}=\frac{1}{K^{\prime }}\left[\begin{array}{c} \sum_{k=1}^{K^{\prime }}\left(ToA_{1,1k}^{\prime }-ToA_{2,1k}^{\prime }\right)\\ \sum_{k=1}^{K\prime }\left(ToA_{1,2k}^{\prime }-ToA_{2,2k}^{\prime }\right)\\ \ldots \\ \sum_{k=1}^{K^{\prime }}\left(ToA_{1,Jk}^{\prime }-ToA_{2,Jk}^{\prime }\right) \end{array}\right]$$

The OPR signal of S_0_ is denoted by *T*_0_’(*k*), and it is expressed as

(13) $${T_{0}}^{\prime }(k)={q_{0}}^{\prime }(nt_{k})=\left[\begin{array}{cccc} T_{01}^{\prime } & T_{02}^{\prime } & \ldots & T_{0K^{\prime }}^{\prime } \end{array}\right]$$

From Equations (10) and (12), the measured ToA difference Δ*ToA_j_* for the same blade at S_1_ can be calculated as

(14) $$\Delta ToA_{j}=(\Delta To{A_{12}}^{\prime }(j)-\Delta ToA_{12}(j))\times t_{k}$$

In summary, the BTT measurement error can be characterized as

(15) $$\Delta =\Delta ToA_{j}-\Delta t_{j}$$

### 2.3. Error Source Analysis

From Equation (3), the error of the theoretical ToA value is calculated as

(16) $$\frac{\Delta (\Delta t_{j})}{\Delta t_{j}}=\frac{\Delta \alpha }{\alpha }-\frac{\Delta \omega }{\omega }$$

The accuracy of Δ*t_j_* directly influences the calibration accuracy of the ToA. In this paper, an error model is constructed based on the calibration model to analyze the sources of error in *α* (changed circumferential angle) and *ω* (rotor angular velocity).

**Figure 4:**
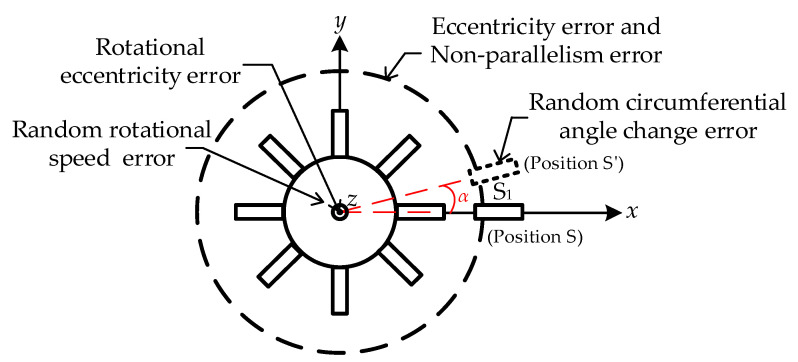
Calibration method error sources distribution.

Specifically, random errors in circumferential angle changes introduce inaccuracies during rotation. Eccentricity errors in the *x* and *y* directions, as well as non-parallelism errors in the *z* direction, collectively result in deviations between the theoretical rotation angle *α* and the actual rotation angle *β* of the tested BTT sensor S_1_. Furthermore, discrepancies between *β* and the measured angle *γ* arise due to eccentricity between the theoretical and actual centers of rotation. For rotor angular velocity *ω*, random error occurs during blade disk rotation, further affecting calibration accuracy. Figure 4 illustrates the distribution of these error sources. This section specifically focuses on systematic errors, including eccentricity error, non-parallelism error, and rotational eccentricity error, which significantly impact the calibration model.

**Figure 5:**
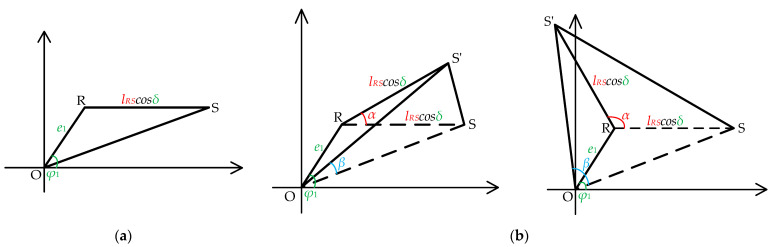
Eccentricity and non-parallel error model: (**a**) before the circumferential angle change in the tested BTT sensor; (**b**) after the circumferential angle change in the tested BTT sensor.

To analyze the effects of eccentricity error and non-parallelism error, an error model incorporating both eccentricity and non-parallelism is constructed, as shown in Figure 5. In this model, the rotation center is denoted as *O*, the angle change center as *R*, the initial position of S_1_ as *S*, and its position after rotation as *S′*. The distance between *S* and *R* is represented by *l_RS_*. The eccentricity angle caused by the angle change is *φ*_1_, the eccentricity magnitude is *e*_1_, and the non-parallel angle formed by non-parallelism error is *δ*. Since the initial positions of the rotation center and angle change center are unknown, the eccentricity error angle *φ*_1_ lies within the range of [−π,π]. Figure 5 illustrates the relative position between the tested BTT sensor S_1_ and the rotation center O before and after a change in the circumferential angle. Considering the non-parallel error, the relative position is represented in a two-dimensional form. Figure 5a shows the relative position of the tested BTT sensor S_1_ before the angle change, while Figure 5b shows its position after the angle change. Since the angle change range spans [−π, π], two graphs are used to represent the possible relative positions. The figure highlights the effects of eccentricity and non-parallel error on the relative position.

Relative to the rotation center *O*, the relationship between the actual rotation angle *β* and the angle change *α* of S_1_ can be described as follows:

(17) $$\beta =f(\alpha ,l_{RS},e_{1},\varphi_{1},\delta )$$

where *α* and *l_RS_* are known quantities, and *e*_1_, *φ*_1_, and *δ* are introduced errors.

As shown in Figure 5, the actual distance between *S* and *R*, influenced by the non-parallelism error *δ*, can be expressed as *l_RS_* cos*δ*. Additionally, the distance *l_OS_* between *S* and *O* is calculated as

(18) $$l_{OS}=\sqrt{{e_{1}}^{2}+{\left(l_{RS}\cos\delta \right)}^{2}+2e_{1}(l_{RS}\cos\delta )\cos\varphi_{1}}$$

where *α* represents the theoretical angle change, *l_RS_* is the initial distance between *S* and *R*, and *e*_1_, *φ*_1_, and *δ* are introduced error sources. After the angle changes by *α*, the tested BTT sensor S_1_ moves to position *S′*, and the distance *l_SS′_* between the initial and final positions is given by

(19) $$l_{SS^{\prime }}=l_{RS}\cos\delta \sqrt{2(1-\cos\alpha )}$$

The updated distance between *S*_1_ and *O* becomes *l_OS′_*, as shown in

(20) $$l_{OS\prime }=\sqrt{{e_{1}}^{2}+{\left(l_{RS}\cos\delta \right)}^{2}+2e_{1}(l_{RS}\cos\delta )\cos(\alpha -\varphi_{1})}$$

From Equations (18)–(20), the actual rotation angle *β* of *S*_1_ in Equation (17) is calculated as

(21) $$\begin{array}{l} \beta =f(\alpha ,l_{RS},e_{1},\varphi_{1},\delta )=\arccos\frac{{l_{OS}}^{2}+{l_{OS^{\prime }}}^{2}-{l_{SS^{\prime }}}^{2}}{2l_{OS}l_{OS^{\prime }}}\\ =\arccos\frac{{e_{1}}^{2}+{\left(l_{RS}\cos\delta \right)}^{2}\cos\alpha +e_{1}(l_{RS}\cos\delta )(\cos\varphi_{1}+\cos(\alpha -\varphi_{1}))}{\sqrt{{e_{1}}^{2}+{\left(l_{RS}\cos\delta \right)}^{2}+2e_{1}l_{RS}\cos\delta \cos\varphi_{1}}\sqrt{{e_{1}}^{2}+{\left(l_{RS}\cos\delta \right)}^{2}+2e_{1}l_{RS}\cos\delta \cos(\alpha -\varphi_{1})}} \end{array}$$

The combined effect of eccentricity and non-parallelism errors is reflected in the discrepancy between the actual rotation angle *β* and the theoretical angle *α*, expressed as *β* − *α*.

**Figure 6:**
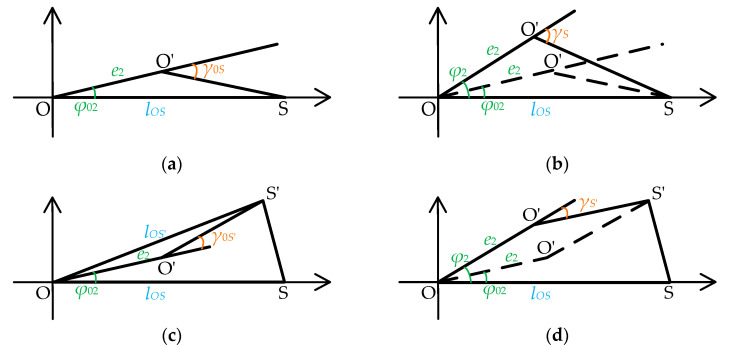
Rotation process with rotational eccentricity error: (**a**) before the circumferential angle of the tested BTT sensor changes, the blade disk does not rotate; (**b**) before the circumferential angle of the tested BTT sensor changes, the blade disk rotates; (**c**) after the circumferential angle of the tested BTT sensor changes, the blade disk does not rotate; (**d**) after the circumferential angle of the tested BTT sensor changes, the blade disk rotates.

Ulteriorly, the above eccentricity and non-parallel error are introduced into the rotational eccentricity error existing in the theoretical rotation center *O* and the actual rotation center *O′*. Based on Figure 4 and Figure 5, a schematic diagram of the rotation process with rotational eccentricity error is constructed, as shown in Figure 6. In this diagram, the rotation process is classified into four states depending on whether BTT sensor circumferential angle changes and the blade disk rotates. Let *O* represent the theoretical center of rotation and *O′* the actual center of rotation. The rotational eccentricity angle is denoted as *φ*_2_ (initially *φ*_02_), and the eccentricity value is *e*_2_. During the rotation process, the point *O′* moves around *O* with a radius of *e*_2_. When the rotation angle changes by Δ*φ* = *φ*_2_ − *φ*_02_, the measurement angle of S_1_ at position *S* is *γ_S_* (initially *γ*_0*S*_), and at position *S’* is *γ_S_’* (initially *γ*_0*S*_*’*). Figure 6a,b illustrate the relative position of the tested BTT sensor S_1_ when its angle remains unchanged, regardless of whether the blade disk rotates. Figure 6c,d depict the relative position of the blade disk, rotating or not, after the tested BTT sensor S_1_ undergoes an angle change.

**Figure 7:**
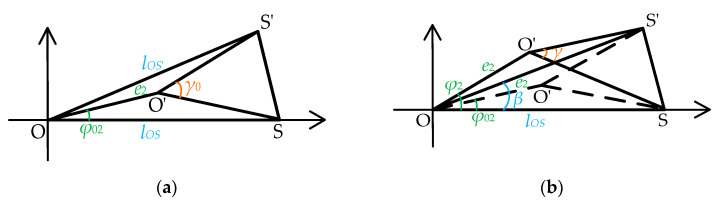
Rotational eccentricity error model: (**a**) the blade disk does not rotate; (**b**) the blade disk rotates.

Combining Figure 6a–d, the relationship between the rotation angle *β* of *S*_1_ and the measurement angle *γ* is illustrated in Figure 7. Specifically, Figure 6a,c are combined to form Figure 7a, while Figure 6b,d are combined to form Figure 7b. By consolidating these cases, the mathematical relationship between the relative positions becomes clearer. Figure 7a represents the relative position without rotation, while Figure 7b represents the relative position of the rotation.

And the relationship can be expressed as

(22) $$\gamma =g(\beta ,l_{OS},e_{2},\varphi_{2})$$

As shown in Figure 7, it can be obtained from the geometric relationship in Δ*O′SS′* that

(23) $$l_{O^{\prime }S}=\sqrt{{e_{2}}^{2}+{l_{OS}}^{2}-2e_{2}l_{OS}\cos\varphi_{2}}$$

(24) $$l_{O^{\prime }S^{\prime }}=\sqrt{{e_{2}}^{2}+{l_{OS^{\prime }}}^{2}-2e_{2}l_{OS^{\prime }}\cos\left|\varphi_{2}-\beta \right|}$$

Ulteriorly, the measurement angle *γ* is determined by Equations (19), (23), and (24).

(25) $$\begin{array}{l} \gamma =g(\beta ,l_{OS},e_{2},\varphi_{2})=\arccos\frac{{l_{O^{\prime }S}}^{2}+{l_{O^{\prime }S^{\prime }}}^{2}-{l_{SS^{\prime }}}^{2}}{2l_{O^{\prime }S}l_{O^{\prime }S^{\prime }}}\\ =\arccos\frac{2{e_{2}}^{2}+{l_{OS}}^{2}+{l_{OS^{\prime }}}^{2}-2e_{2}(l_{OS}\cos\varphi_{2}+l_{OS^{\prime }}\cos\left|\varphi_{2}-\beta \right|)-2{l_{RS}}^{2}\cos^{2}\delta (1-\cos\alpha )}{2\sqrt{\left({e_{2}}^{2}+{l_{OS}}^{2}-2e_{2}l_{OS}\cos\varphi_{2})({e_{2}}^{2}+{l_{OS^{\prime }}}^{2}-2e_{2}l_{OS^{\prime }}\cos\left|\varphi_{2}-\beta \right|\right)}} \end{array}$$

Finally, the rotational eccentricity error is expressed as the discrepancy *γ*−*β*, as shown in Equation (25).

By combining Equations (21) and (25), the theoretical rotation angle *α* and the actual measurement angle *γ* can be linked through the intermediate variable *β*, as follows:

(26) $$\gamma =g(f(\alpha ,l_{RS},e_{1},\varphi_{1},\delta ),h(l_{RS},e_{1},\varphi_{1},\delta ),e_{2},\varphi_{2})$$

From Equation (26), the relationship between *γ*, *α*, and *β* is established. The eccentricity error, non-parallelism error, and rotational eccentricity error contribute to the overall discrepancy expressed as *γ*-*α*.

In summary, the error of the theoretical ToA value is calculated as

(27) $$\frac{\Delta (\Delta t_{j})}{\Delta t_{j}}=\frac{g(f(\alpha ,l_{RS},e_{1},\varphi_{1},\delta ),h(l_{RS},e_{1},\varphi_{1},\delta ),e_{2},\varphi_{2})-\alpha }{\alpha }-\frac{\Delta \omega }{\omega }$$

The specific quantitative analysis of this error will be rigorously evaluated in the uncertainty analysis described in Section 3.

## 3. Blade-Tip-Timing Accuracy Evaluation Device

### 3.1. Device Design

**Figure 8:**
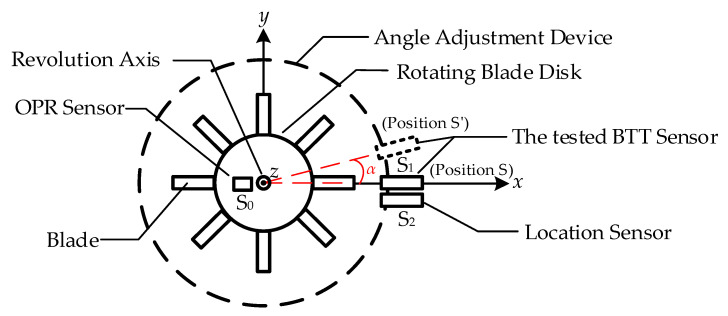
BTT accuracy evaluation device model.

Based on the theoretical model described above, this paper develops a BTT accuracy evaluation device, as shown in the model diagram in Figure 8. The devices in Figure 8 correspond with the functional requirements in Figure 3. The device utilizes a high-precision angle adjustment mechanism to induce a controllable circumferential angle change in the BTT sensor. Coupled with the high-stability rotating blade disk, the device measures the time at which the BTT sensor reaches the same blade at different circumferential positions. The rotating blade disk is coaxial with the angle adjustment device, and the tested BTT sensor S_1_ rotates synchronously with the angle adjustment mechanism, with its probe facing the blade end. Sensors S_0_ and S_2_, respectively, serve as the OPR sensor and location sensor. The S_0_ probe is oriented towards the shaft, while the S_2_ probe faces the blade end. Both sensors are fixed in position.

The selection and design of the device directly impact the test accuracy and results. Based on Section 2, the angle adjustment device is selected, the rotating blade disk test bench is constructed, and the device’s connection and positioning components are designed. Both the circumferential angle change error of the angle adjustment device and the rotational speed error of the rotating blade disk significantly influence the measurement of ToA. As a traceability device for the angular displacement change in the BTT sensor, the angle adjustment device must be highly accurate and capable of achieving precise angular adjustments. Additionally, the device must have sufficient load capacity to ensure that the BTT sensor rotates synchronously with the adjustment mechanism. To minimize the influence of Abbe error, the axis of the angle adjustment device should align with the axis of the rotating blade disk. As the signal source in the BTT measurement system, the blade disk must have minimal speed fluctuation to reduce random rotational speed error. Finally, during the test, the blade should remain stable and avoid vibration to ensure that ToA measured by the BTT sensor is solely influenced by the angle change from the angle adjustment device.

The angle adjustment device is selected as a multi-tooth indexing table, which conforms to national metrological verification standards and is capable of calibration and measurement. The maximum indexing interval error does not exceed 0.2″ (equivalent to 5.556 × 10^−5^°) and supports a load capacity of 20 kg. For the rotating blade disk test bench, an air-floating turntable is chosen due to its advantages of zero friction and minimal vibration. Speed fluctuations are controlled within ±0.01 rpm at 1000 rpm. The blade disk is designed with an integrated, short blade structure, ensuring minimal vibration at the test speed. A convex feature is integrated into the shaft of the blade disk to facilitate coaxial positioning and trigger the OPR sensor. Based on Equations (3) and (16), with an angle change of 0.5° and a rotation speed of 1000 rpm, the error introduced by the angle adjustment device and the rotating blade disk test bench is ±0.01009 μs, and the relative accuracy is ±0.01211%. These values meet the required measurement specifications.

**Figure 9:**
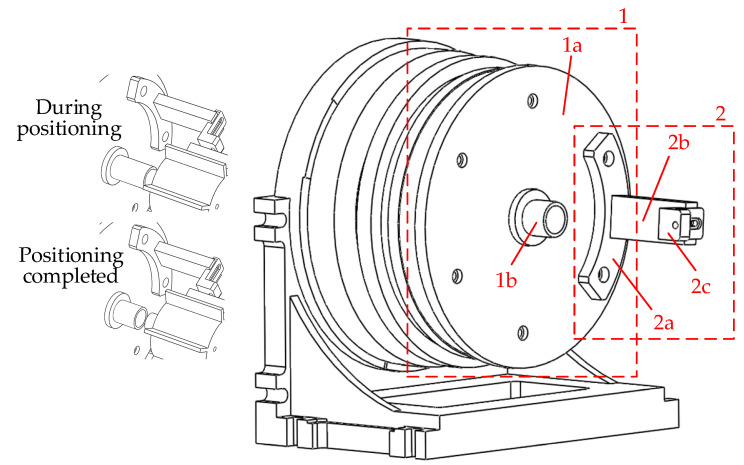
Coaxial positioning device and the tested BTT sensor fixture, where 1—coaxial positioning device; 1a—connection part; 1b—coaxial positioning adjustable part; 2—the tested BTT sensor fixture; 2a—fixing part; 2b—blade proximity part; 2c—clamping part.

As a connecting device, the coaxial positioning and sensor fixing device must ensure that the multi-tooth indexing table, the rotating blade disk test bench, and the rotation center of the tested BTT sensor are coaxial. Additionally, the tested BTT sensor, location sensor, and OPR sensor must be stable and reliable. To achieve coaxial alignment and secure the BTT sensor, the coaxial positioning device and the tested BTT sensor fixture are designed, as shown in Figure 9. One end of the coaxial positioning device is fixed to the rotating surface of the multi-tooth indexing table, while the other end can be adjusted along the axis. During positioning, the coaxial positioning adjustable part extends to make contact with the positioning bulge of the blade disk, aligning the multi-tooth indexing table with the rotating blade disk test bench. Once positioning is complete, the part contracts, ensuring coaxial alignment without interfering with the blade disk rotation. The tested BTT sensor fixture is fixed at one end to the coaxial positioning device. Its arc extension circle is concentric with the coaxial positioning device, ensuring that the rotation center of the tested BTT sensor aligns with that of the multi-tooth indexing table. The other end of the device extends to the blade end, allowing for adjustment of the tip clearance.

**Figure 10:**
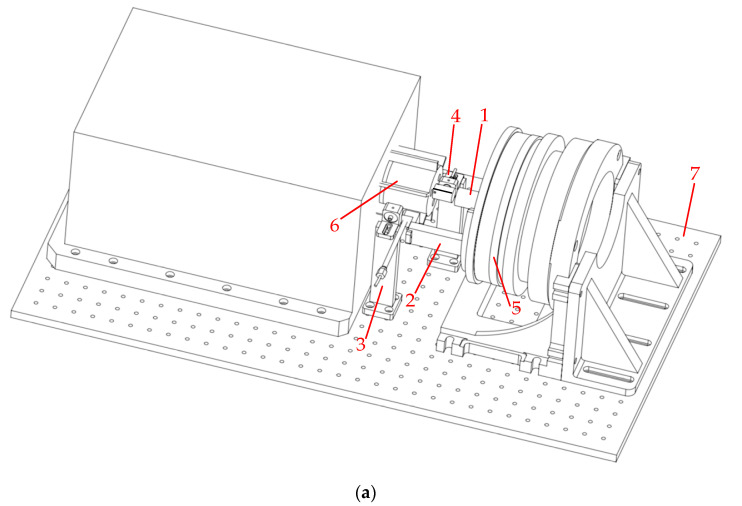

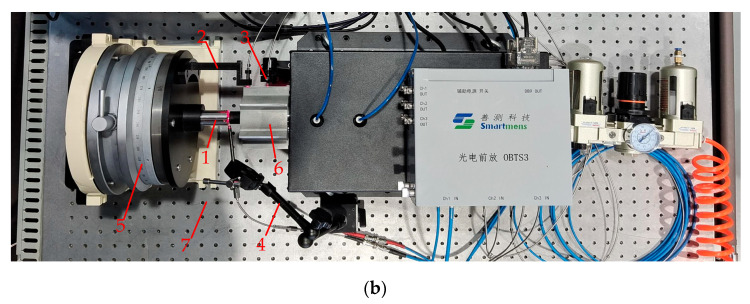
BTT accuracy evaluation device, where 1—coaxial positioning device; 2—the tested BTT sensor fixture; 3—location sensor fixture; 4—OPR sensor fixture; 5—multi-tooth dividing table; 6—rotating blade disk test bench; 7—optical platform: (**a**) overall structure; (**b**) physical object.

Finally, clamping devices for the location sensor and OPR sensor are also designed. The overall structure and physical diagrams are shown in Figure 10.

### 3.2. Uncertainty Evaluation of Device Based on Monte Carlo Method

As discussed in Section 2.3, the eccentricity and non-parallelism errors between the multi-tooth indexing table and the rotating blade disk, along with rotational eccentricity errors, are nonlinear and exhibit multivariate correlations. Therefore, this paper employs the Monte Carlo (MCM) method to evaluate measurement uncertainty, which offers several advantages: it does not require linearity in the uncertainty analysis model, does not assume a symmetrical distribution for input data, is unaffected by input correlations or model complexity, and does not require assumptions about the distribution of the measured data.

The core of establishing the measurement uncertainty analysis model is to identify and quantify the sources of uncertainty. Based on error analysis and the device structure, these sources are categorized as follows: Indication error of the multi-tooth indexing table and speed fluctuation error of the rotating blade disk test bench reflect system errors that impact the measurement results. Blade machining and installation errors can induce rotational eccentricity. The positioning and fixing device accounts for the impact of dimensional and geometric tolerances on measurement results during processing and installation. Specifically, it affects the coaxiality and non-parallelism between the multi-tooth indexing table, the tested BTT sensor, and the blade disk, leading to mechanical errors in the measurement path. In addition, measurement repeatability, environmental fluctuations, and random noise also affect the measurement results.

To address the influence of each uncertainty source, the uncertainty analysis model of the BTT accuracy evaluation device is derived from Equation (3), as expressed below:

(28) $$\Delta t_{j}=\frac{(\alpha_{2}-\alpha_{1})+\theta_{E}+\theta_{ie}+\theta_{inp}+\theta_{re}+\theta_{mr}}{\frac{2\pi (n_{r}+n_{rsf}+n_{mr})}{60}}+\varepsilon_{rn}$$

where *α*_1_ is the initial angle of the multi-tooth indexing table, *α*_2_ is the angle of the multi-tooth indexing table after angular change, *θ_E_* is the uncertainty component due to indication error of the multi-tooth indexing table, *θ_ie_* is the uncertainty component due to eccentricity from inhomogeneity of the coaxial positioning device and BTT sensor fixture, *θ_inp_* is the uncertainty due to non-parallelism from inhomogeneity of the coaxial positioning device and BTT sensor fixture, *θ_re_* is the uncertainty component due to rotor rotational eccentricity, *θ_mr_* is the uncertainty component from repeatability of angle measurement of the multi-tooth indexing table, *n_r_* is the rotor rotational speed, *n_rsf_* is the uncertainty component from rotor rotational speed fluctuations, *n_mr_* is the uncertainty component from rotational speed measurement repeatability during rotor rotation, and *ε_m_* is the uncertainty component from environmental fluctuations (e.g., temperature, random noise) during testing.

Among the uncertainty components, *θ_ie_* and *θ_inp_* affect the actual rotation angle of S_1_, while *θ_re_* influences the measured angle of S_1_. Combined with Section 2.3, it can be expressed as

(29) $$\begin{array}{l} \theta_{ie}+\theta_{inp}=\beta -\alpha \\ \theta_{re}=\gamma -\beta \end{array}$$

Therefore, Equation (28) can be further expressed as

(30) $$\Delta t_{j}=\frac{(\alpha_{2}-\alpha_{1})+\theta_{E}+(\gamma -\alpha )+\theta_{mr}}{\frac{2\pi (n_{r}+n_{rsf}+n_{mr})}{60}}+\varepsilon_{rn}=\frac{g(f(\alpha ,l_{RS},e_{1},\varphi_{1},\delta ),h(l_{RS},e_{1},\varphi_{1},\delta ),e_{2},\varphi_{2})+\theta_{E}+\theta_{mr}}{\frac{2\pi (n_{r}+n_{rsf}+n_{mr})}{60}}+\varepsilon_{rn}$$

Based on Equation (30), the probability distributions and corresponding parameters of each input are analyzed. For the multi-tooth indexing table, the average angle variation *α* after multiple measurements is 0.5°, with the standard deviation of 0°. Since the test method uses differential measurement, the mean and standard deviation of the uncertainty component *θ_E_* and *θ_mr_* are both 0°. For the positioning and fixing devices, based on the structural design, the tolerance of the distance *l_R__S_* between the tested BTT sensor S_1_ and the center of the multi-tooth indexing table is controlled within ±0.02 mm, ranging from 43.48 to 43.52 mm. The eccentricity magnitude *e*_1_, affected by the coaxial positioning device, introduces a measurement uncertainty component in the range of [−0.01, 0.01] mm. The eccentricity angle *φ*_1_ can range from −180 to 180°, as the angle position cannot be determined during rotation. The non-parallel angle *δ*, primarily affected by component installation, introduces an uncertainty component in the range of [−0.1, 0.1]°, based on empirical values. These probability distributions are assumed to be uniformly distributed. For the rotating blade disk test bench, after dynamic balancing, the measurement uncertainty component introduced by the eccentricity magnitude *e*_2_ (due to blade disk processing and installation errors) ranges from −0.003 to 0.003 mm. The eccentricity angle *φ*_2_ is the same as *φ*_1_, also ranging from −180 to 180°. Both distributions are uniformly distributed. The set rotational speed *n_r_* is 1000 rpm. After 10 tests, the average value is 1000.3345 rpm, with the standard deviation of 2.249 × 10^−4^ rpm. The uncertainty component *n_mr_*, introduced by the repeatability of rotational speed measurement, is derived accordingly. The uncertainty component *n_rsf_*, due to blade speed fluctuations, ranges from −0.01 to 0.01 rpm and is also uniformly distributed. Finally, in the laboratory environment, after isothermal conditions are achieved, the test duration is short. In this brief period, the measurement environment and noise fluctuations in the measurement system are assumed stable, so the uncertainty component *ε_m_*, caused by circuit noise and ambient temperature, can be neglected. In summary, the probability distributions of each input variable are summarized in Table 1.

**Figure 11:**
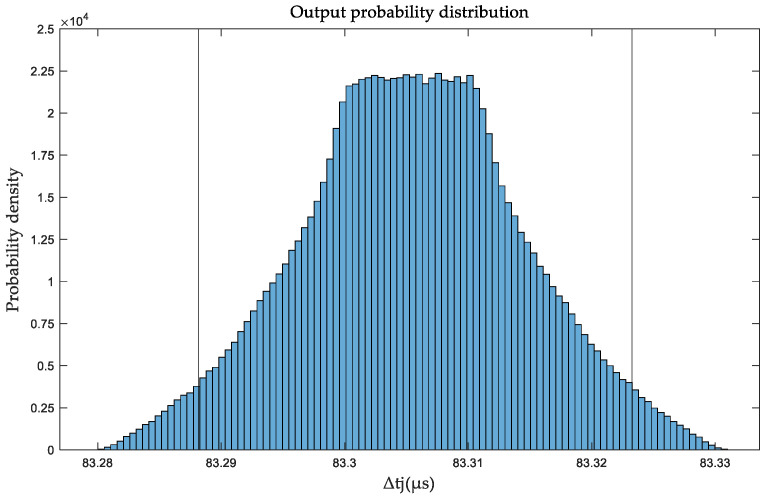
The probability distribution of ToA theoretical value Δ*t_j_*.

Based on Table 1, the MCM is used to evaluate the uncertainty of the BTT accuracy evaluation device. The probability distribution of the output, represented by the theoretical ToA value Δ*t_j_*, is shown in Figure 11. The results indicate that at 0.5° and 1000 rpm, the estimated value is 83.3055 μs, with the standard uncertainty of 8.824 × 10^−3^ μs. The 95% confidence interval, indicated by the two vertical lines, is [83.2881, 83.3233] μs. The figure demonstrates that the error follows a trapezoidal distribution.

## 4. Blade-Tip-Timing Accuracy Evaluation Test

### 4.1. Test Scheme and Measurement Data

In the previous sections, the feasibility of the proposed method and device was evaluated through error analysis and uncertainty evaluation. This section focuses on evaluating the accuracy of the optical fiber BTT measurement system, which has been widely applied in the industry. By adjusting the angle of the multi-tooth indexing table and the speed of the rotating blade disk test bench, simulations of various blade vibration displacements and speed conditions are conducted.

The specific test plan and average results are shown in Table 2. Conditions 1–11 simulate different blade vibration displacements, while Conditions 1–2 and 12–25 simulate different rotational speeds. Specifically, subtracting Condition 1 from Conditions 2–11 allows for the simulation of varying blade vibration displacements at a constant rotational speed. Similarly, pairing and subtracting Conditions 1–2 and 12–25 enables the simulation of different rotational speeds under the same blade vibration displacement. In Condition 1, with the multi-tooth indexing table rotation angle *α* set to 0°, an initial angle difference is observed between the tested BTT sensor S_1_ and the location sensor S_2_. Each test condition was repeated 20 times, with 50 laps per test. The average measurement results of 20 times and 8 blades under different conditions were obtained. The ToA difference measurement Δ*ToA*_12_ between S_1_ and S_2_, as well as the rotational speed measurement *n_rm_* from the OPR sensor S_0_, were recorded under various rotation angles and speeds, and the angle difference measurement *α_m_*_12_ was calculated. Among them, the angle difference measurement, derived from the ToA difference measurement and rotational speed measurement, is used as the result indicator. On the one hand, the subsequent angle measurements can be directly compared with the rotation angles of the multi-tooth indexing table, providing a more intuitive assessment. On the other hand, as shown in Table 2, the speed fluctuation of the rotating test bench is controlled within ±0.01 rpm, although some systematic error remains. The rotational speed is measured by the OPR sensor.

**Figure 12:**
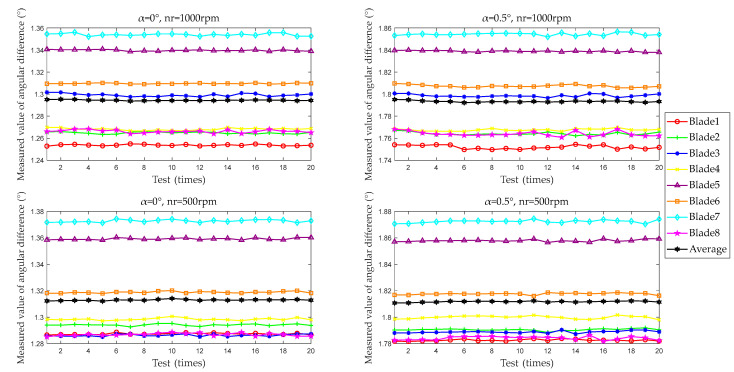
Single test results for a single blade (examples from Conditions 1, 2, 12, and 13).

The test results for a single blade are shown in Figure 12, using Conditions 1, 2, 12, and 13 as examples. In each condition, differences in the angle difference measurements of different blades are observed. These variations are attributed to differences in blade length and angular distribution during the manufacturing process. Comparing Conditions 1 and 12, differences are also noted in the measured values of the same blade at different speeds. These discrepancies arise from variations in data processing within the BTT measurement system at different speeds. Signal noise and sampling rate of the BTT measurement system lead to differences in ToA obtained through different noise reduction and ToA extraction methods. The method and device proposed in this paper rely on the rotation angle of the multi-tooth indexing table. The OPR sensor distinguishes different blades, while the location sensor handles differential processing. The rotational speed is controllable, and fluctuations are negligible, ensuring that the aforementioned differences do not impact the test results.

To simulate different vibration displacement, the angle difference measurement values from the BTT measurement system, using the multi-tooth indexing table’s rotation angle as the reference, are obtained by subtracting the test results of any two conditions at the same rotational speed. The test results for Conditions 2–11 are sequentially subtracted from those of Condition 1, yielding average test results for the multi-tooth indexing table rotation angles of 0.5°, 1°, 1.5°, …, 20° at a constant rotational speed of 1000 rpm, as shown in Table 3.

To simulate different rotational speed conditions, the average test results are obtained by varying the speed of the blade disk while maintaining a rotation angle difference of 0.5° for the multi-tooth indexing table across working Conditions 1, 2, and 12–25. The results are shown in Table 4.

As seen above, the relative deviation of the average test results reaches −0.6347%, while the relative deviation typically required is less than 1%. By simulating various vibration displacement and rotational speed conditions, it is further confirmed that the BTT accuracy evaluation method and device proposed in this paper effectively assess the BTT measurement system’s accuracy.

### 4.2. System Error Compensation of BTT Measurement System

**Figure 13:**
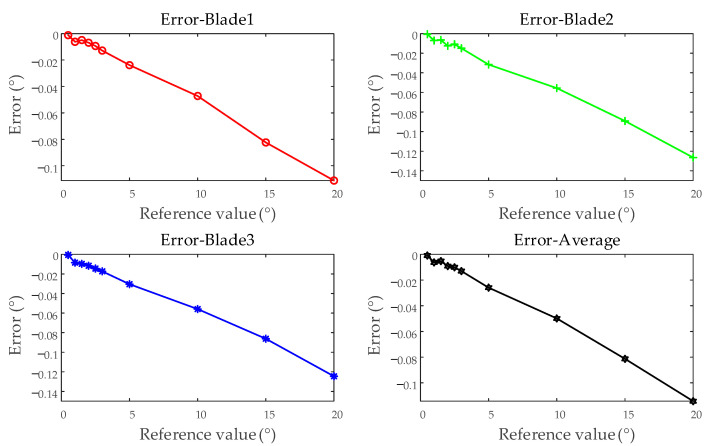
Single test results for a single blade (examples from blades 1, 2, 3, and the average of all blades).

Select the first five tests simulating different vibration displacements to calculate the error, as shown in Figure 13. Using blades 1, 2, and 3, along with the average of all blades, as examples, a linear relationship is observed between the errors and the reference values. This section will apply linear correction to the system error of the BTT measurement system.

**Figure 14:**
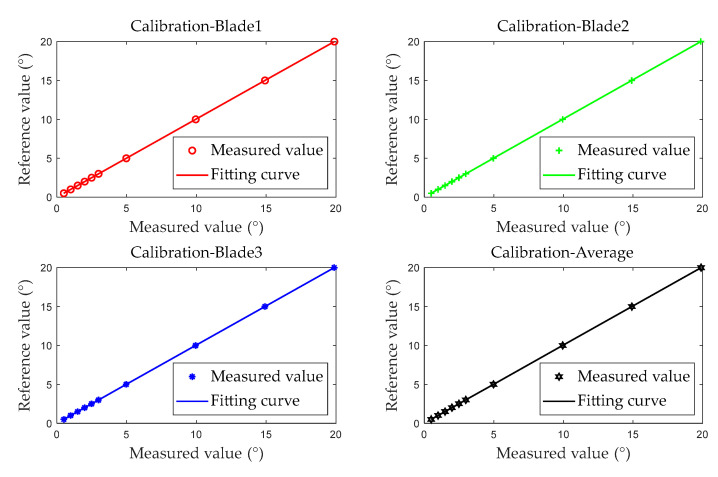
Calibration fitting (examples from blades 1, 2, 3, and the average of all blades).

The first five test results are used as calibration data to generate the calibration fitting curve, as shown in Figure 14.

Building upon the polynomial fitting in Equation (4), the calibration fitting curve is expressed as follows:

(31) $$Val_{\Delta t}=a_{1}Val_{\Delta ToA}+a_{0}$$

where *Val*_Δ*ToA*_ is the measured value of the BTT measurement system, and *Val*_Δ*t*_ is the reference value of the BTT accuracy evaluation device. The linear correction coefficients for the system error of the BTT measurement system are presented in Table 5. The coefficient of determination *R*^2^ for the calibration curve exceeds 0.999999, indicating that the linear fitting meets the calibration requirements.

**Figure 15:**
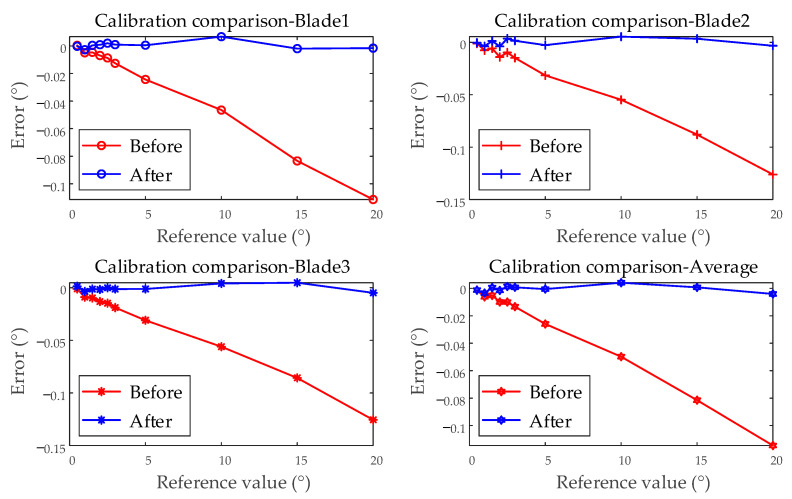
Comparison before and after correction (examples from single test; blades 1, 2, 3; and the average of all blades).

Using the linear correction coefficient for system error, the remaining 15 tests are corrected. The comparison before and after correction is shown in Figure 15. The linear component of the error is corrected. Before the correction, the maximum error is −0.1260°, and the relative accuracy is −0.8935%. After the correction, the maximum error is reduced to 5.389 × 10^−3^°, and the relative accuracy improves to 0.3735%. The BTT accuracy evaluation device achieves linear system error compensation for the BTT measurement system.

### 4.3. Accuracy Evaluation of BTT Measurement System

In this section, the accuracy of the optical fiber BTT measurement system is evaluated using four indicators: error, relative accuracy, standard deviation, and relative standard deviation. Error and relative accuracy serve as direct evaluation indices for assessing the system’s accuracy. Standard deviation and relative standard deviation are used as repeatability indices to evaluate the dispersion of measurement values and the stability of the measurement system.

**Figure 16:**
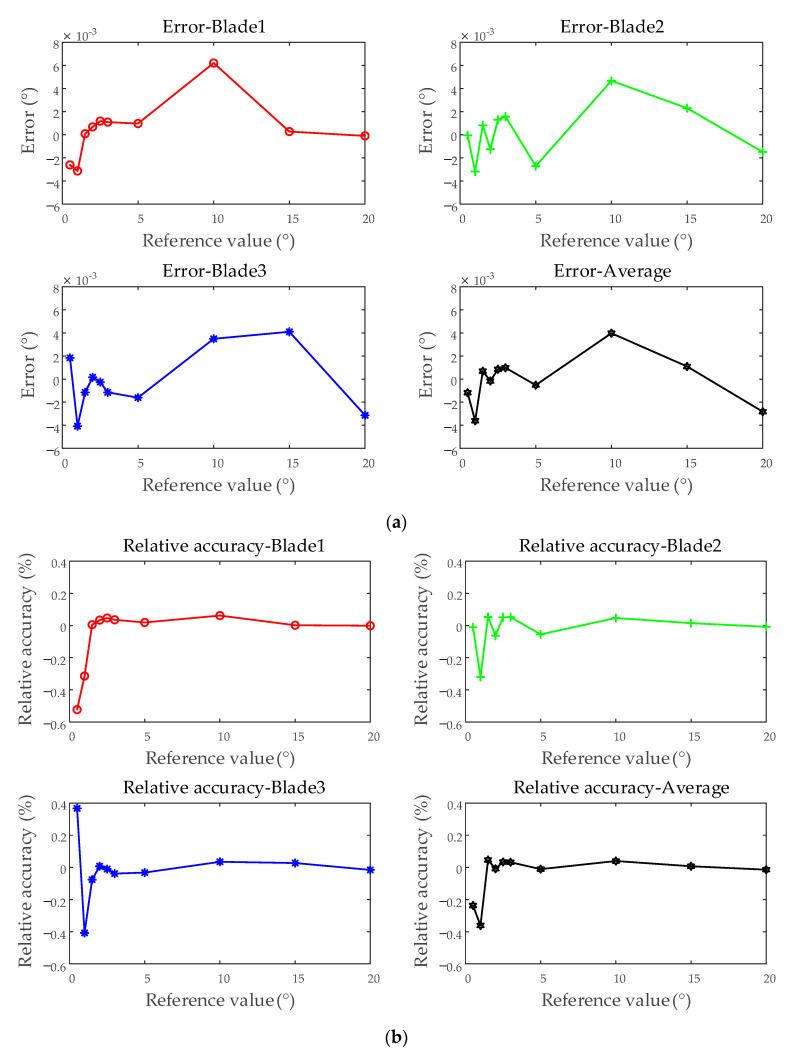

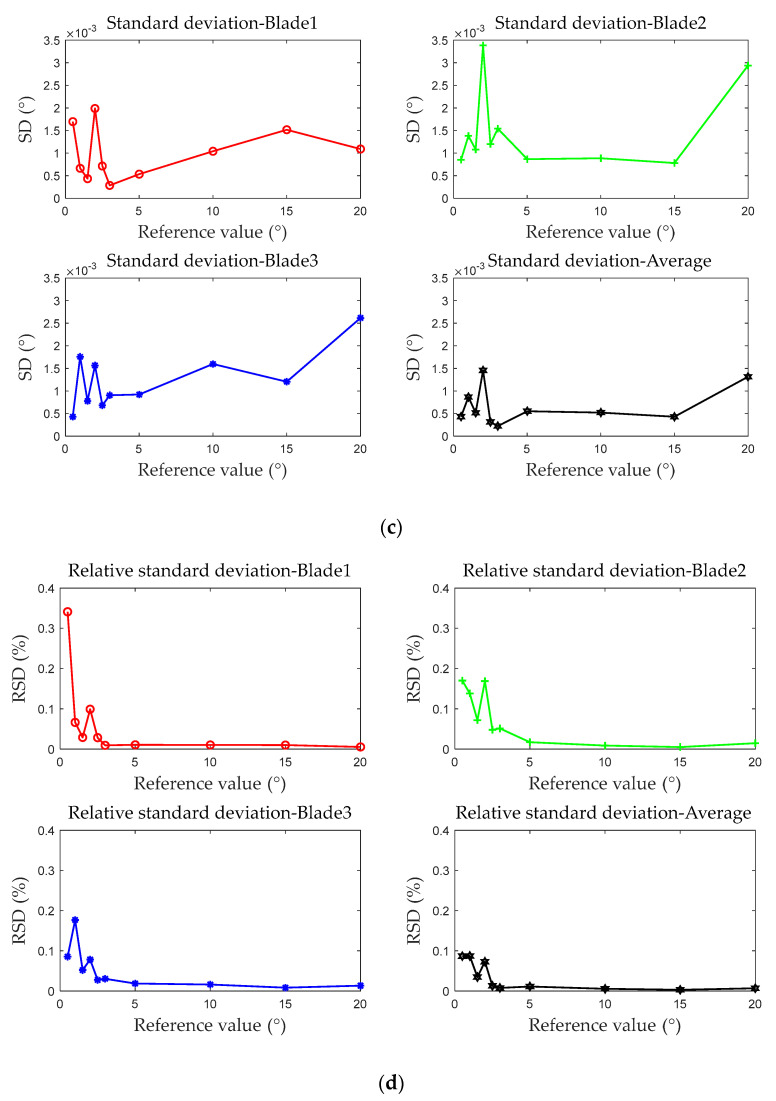
Accuracy evaluation of the BTT measurement system—simulating different vibration displacements: (**a**) error; (**b**) relative accuracy; (**c**) standard deviation; (**d**) relative standard deviation.

Under different vibration displacement conditions, the test results of average blades under each test were compared. The maximum error observed was −5.636 × 10^−3^°, while the minimum error was −2.219 × 10^−8^°, with an overall range of −5.636 × 10^−3^° to 4.882 × 10^−3^°. The minimum relative accuracy recorded was −0.5636%, and the maximum was −1.480 × 10^−7^%, spanning from −0.5636% to 0.1851%. The standard deviation varied between 2.232 × 10^−4^° and 3.422 × 10^−3^°, while the relative standard deviation ranged from 1.903 × 10^−3^% to 0.3646%. As shown in Figure 16, the accuracy evaluation results under simulated vibration displacement conditions are presented, using blades 1, 2, and 3, as well as the average of all blades, as examples. And the error and relative accuracy are illustrated based on the average test results. The figure illustrates that, compared to individual blade test results, the average of all blades exhibits smaller errors and higher repeatability accuracy. This improvement is attributed to the increased amount of test data, which helps mitigate the influence of random measurement system errors.

**Figure 17:**
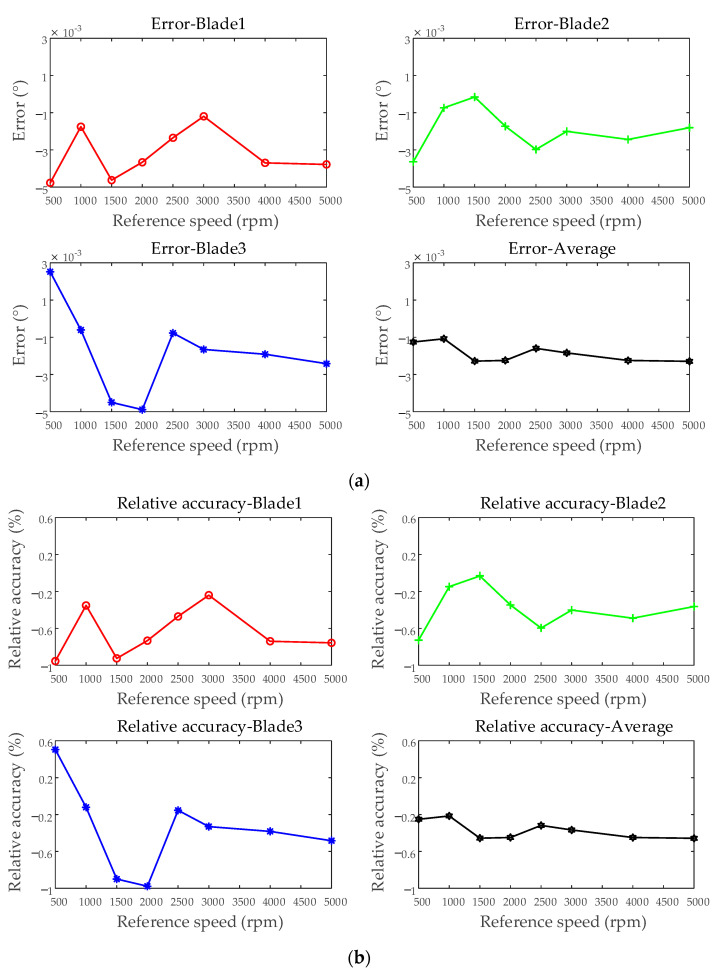

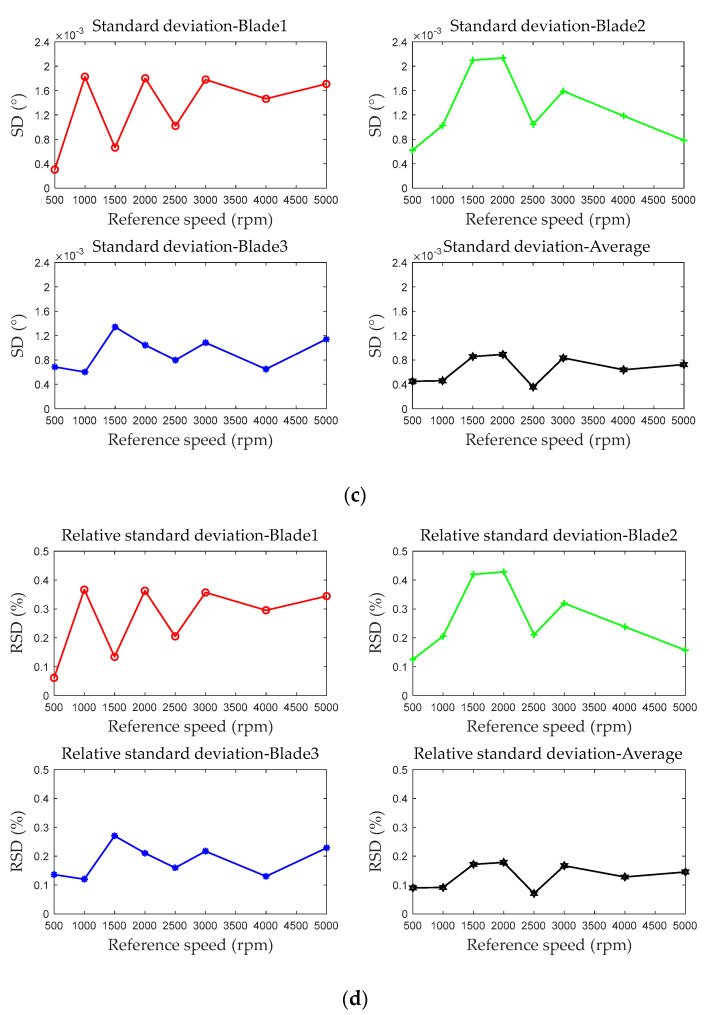
Accuracy evaluation of the BTT measurement system—simulating different rotational speeds: (**a**) error; (**b**) relative accuracy; (**c**) standard deviation; (**d**) relative standard deviation.

Under different rotational speed conditions, the test results of average blades under each test were compared. The maximum error recorded was −3.738 × 10^−3^°, while the minimum error was 3.176 × 10^−5^°, with an overall range of −3.738 × 10^−3^° to 3.176 × 10^−5^°. The minimum relative accuracy observed was −0.7476%, and the maximum was 6.351 × 10^−3^%, spanning from −0.7476% to 6.351 × 10^−3^%. The standard deviation varied between 3.402 × 10^−4^° and 2.413 × 10^−3^°, while the relative standard deviation ranged from 6.824 × 10^−2^% to 0.4855%. Figure 17 presents the accuracy evaluation results under different rotational speed conditions. The variations in test results at different rotational speeds are primarily attributed to the influence of the BTT measurement system’s bandwidth, sampling rate, and ToA extraction algorithm on each index.

The evaluation results show that the relative accuracy of the optical fiber BTT measurement system is better than 0.8%, and the repeatability accuracy is better than 0.5%. Through the evaluation using four types of indicators, the BTT measurement system has been assessed in terms of precision, accuracy, and stability.

## 5. Conclusions

In this paper, a method for evaluating the accuracy of BTT measurement by directly calibrating ToA is proposed, and a BTT accuracy evaluation device is developed to assess the accuracy of BTT measurement system. The main conclusions are summarized as follows:

(1) A ToA direct calibration method is proposed, which equivalently transforms the ToA variation caused by blade vibration into the circumferential angle difference between the BTT sensor and the rotating blade disk, while accounting for the effects of eccentricity, non-parallelism, and other errors. As ToA serves as the fundamental data for the BTT method, the accuracy of the BTT measurement system is evaluated using the ToA obtained through this direct calibration approach.
(2) A BTT accuracy evaluation device is designed. The device incorporates a multi-tooth indexing table, a high-precision turntable, and the necessary fixtures to realize the required functions. Considering both system and random factors, the device’s uncertainty is evaluated using the Monte Carlo method. Under the conditions of 0.5° and 1000 rpm, the estimated uncertainty is 83.3055 μs, with the standard uncertainty of 8.824 × 10^−3^ μs, and the 95% confidence interval of [83.2881, 83.3233] μs.
(3) Accuracy evaluation tests of the BTT measurement system were performed using the developed device. The device simulates varying vibration displacement and rotational speed conditions. The optical fiber BTT system’s measurement accuracy is evaluated across four metrics: error, relative accuracy, SD, and RSD. The relative accuracy of the tested optical fiber BTT system is better than 0.8%, and the RSD is below 0.5%. The results demonstrate that the proposed method and device successfully evaluate the accuracy and stability of the BTT measurement system while directly assessing its ToA.

Future work will focus on optimizing the structural design of the BTT accuracy evaluation device, incorporating more blade disk structures and rotational conditions to better meet industrial testing requirements, thereby enabling accurate evaluation of the BTT measurement system under various operating conditions.

## 6. Patents

Niu, G.; Zhou, Q.; Duan, F.; et al. A device and method for measuring vibration displacement accuracy verification in a blade tip timing vibration measurement system. China: CN202311096563.0, 26 December 2023.

Niu, G.; Zhou, Q.; Duan, F.; et al. A device for verifying vibration displacement measurement accuracy in blade tip timing vibration measurement systems. Tianjin, China: CN202322327594.4, 26 March 2024.

## Figures and Tables

**Table 1:** Probability distributions of input variables.

Input Variables	Distribution Parameters				Probability Distribution
	μ	σ	a	b	
*α*/°	0.5	0	/	/	N(μ, σ^2^)
*l_RS_*/mm	/	/	43.48	43.52	R(a, b)
*e*_1_/mm	/	/	−0.01	0.01	R(a, b)
*φ*_1_/°	/	/	−180	180	R(a, b)
*δ*/°	/	/	−0.1	0.1	R(a, b)
*e*_2_/mm	/	/	−0.003	0.003	R(a, b)
*φ*_2_/°	/	/	−180	180	R(a, b)
*n_r_*/rpm	1000.3345	2.249 × 10^−4^	/	/	N(μ, σ^2^)
*n_rsf_*/rpm	/	/	−0.01	0.01	R(a, b)

**Table 2:** The accuracy evaluation test scheme and average measurement results of the optical fiber BTT measurement system. Working Conditions 2–11 are grouped with Condition 1, respectively, to simulate changes in vibration displacement, while Conditions 1–2, 12–13, 14–15, 16–17, 18–19, 20–21, 22–23, and 24–25 simulate changes in rotational speed.

Test Type	Condition Number	Multi-Tooth Indexing Table Rotation Angle *α*/°	Rotational Blade Disk Set Speed *n_r_*/rpm	ToA Difference Measurement Δ*ToA*_12_/μs	Rotational Speed Measurement *n_r__m_*/rpm	Angle Difference Measurement *α_m_*_12_/°
Simulating vibration displacement changes	1	0	1000	215.6666	1000.3351	1.2944
	2	0.5	1000	298.7925	1000.3346	1.7934
	3	1	1000	381.2203	1000.3342	2.2881
	4	1.5	1000	464.7491	1000.3344	2.7894
	5	2	1000	547.4308	1000.3346	3.2857
	6	2.5	1000	630.4690	1000.3344	3.7841
	7	3	1000	713.3132	1000.3345	4.2813
	8	5	1000	1044.3971	1000.3343	6.2685
	9	10	1000	1873.4700	1000.3344	11.2446
	10	15	1000	2701.3150	1000.3344	16.2133
	11	20	1000	3528.9697	1000.3344	21.1809
Simulating rotational speed changes	12	0	500	437.5131	500.1668	1.3130
	13	0.5	500	603.6859	500.1672	1.8117
	14	0	1500	146.5108	1500.4989	1.3190
	15	0.5	1500	201.7924	1500.5009	1.8167
	16	0	2000	109.5501	2000.6677	1.3150
	17	0.5	2000	151.0234	2000.6701	1.8129
	18	0	2500	87.6196	2500.8351	1.3147
	19	0.5	2500	120.8415	2500.8340	1.8132
	20	0	3000	72.6806	3001.0036	1.3087
	21	0.5	3000	100.3465	3001.0159	1.8068
	22	0	4000	54.9741	4001.3608	1.3198
	23	0.5	4000	75.7061	4001.3923	1.8176
	24	0	5000	44.2745	5002.6730	1.3289
	25	0.5	5000	60.8521	5002.9860	1.8267

**Table 3:** Average test results for simulating different vibration displacements.

Multi-Tooth Indexing Table Rotation Angle α/°	Circumferential Angle Measurement α_m_/°	Error/°	Relative Accuracy/%
0.5	0.4989	−1.079 × 10^−3^	−0.2158
1	0.9937	−6.347 × 10^−3^	−0.6347
1.5	1.4950	−5.006 × 10^−3^	−0.3337
2	1.9913	−8.750 × 10^−3^	−0.4375
2.5	2.4896	−1.035 × 10^−2^	−0.4142
3	2.9869	−1.312 × 10^−2^	−0.4374
5	4.9740	−2.596 × 10^−2^	−0.5191
10	9.9501	−4.985 × 10^−2^	−0.4985
15	14.9189	−8.112 × 10^−2^	−0.5408
20	19.8865	−1.135 × 10^−1^	−0.5677

**Table 4:** Average test results for simulating different rotational speed.

Rotational Blade Disk Set Speed *n_r_*/rpm	Circumferential Angle Measurement α_m_/°	Error/°	Relative Accuracy/%
500	0.4987	−1.314 × 10^−3^	−0.2628
1000	0.4989	−1.079 × 10^−3^	−0.2158
1500	0.4977	−2.298 × 10^−3^	−0.4596
2000	0.4978	−2.152 × 10^−3^	−0.4305
2500	0.4985	−1.507 × 10^−3^	−0.3014
3000	0.4982	−1.8385 × 10^−3^	−0.3677
4000	0.4978	−2.2467 × 10^−3^	−0.4493
5000	0.4977	−2.2922 × 10^−3^	−0.4584

**Table 5:** Linear correction coefficients for system error.

Blade Number	*a*_1_	*a*_0_	*R* ^2^	Blade Number	*a*_1_	*a*_0_	*R* ^2^
1	1.0057	−0.0035	0.99999985	6	1.0058	−0.0016	0.99999991
2	1.0063	−0.0024	0.99999982	7	1.0057	−0.0049	0.99999993
3	1.0061	−0.0006	0.99999984	8	1.0054	−0.0045	0.99999972
4	1.0052	−0.0039	0.99999986	Average	1.0057	−0.0029	0.99999989
5	1.0056	−0.0017	0.99999986				

## Data Availability

All data that support the findings of this study are included within this article.

## Abbreviations

The following abbreviations are used in this manuscript:

BTT	Blade tip timing
ToA	Time of arrival
OPR	Once per revolution
*i* (*i* = 1, 2, …, *I*)	Number of BTT sensors
*j* (*j* = 1, 2, …, *J*)	Number of blades on the blade disk
*y_ij_*	Vibration displacement for different BTT sensors and blades
*t_ij_*	Time for vibrating blade *j* to reach BTT sensor *i*
*t’_ij_*	Time for blade *j* to reach BTT sensor *i* when not vibrating
Δ*t_ij_*	Difference between the time when blade *j* reaches BTT sensor *i* while vibrating and the theoretical ToA
S_1_	The tested BTT sensor
*r*	Rotor radius
*ω*	Rotor angular velocity
*α*	Theoretical angular displacement of S_1_
Δ*t_j_*	Theoretical ToA difference before and after S_1_ rotation
*a_n_*, *a_n_*_-1_, *a_n_*_-2_, *…*, *a*_1,_ *a*_0_	Calibration coefficient
S_0_	OPR sensor
S_2_	Location sensor
*f*(Δ*S*_1_)	ToA difference between before and after S_1_ rotation
*f*(Δ*S*_12_)	ToA difference between S_1_ and S_2_ before S_1_ rotation
*f*(Δ*S*_12_’)	ToA difference between S_1_ and S_2_ after S_1_ rotation
*T*	Initial test duration
*n_r_*	Initial rotational speed
*K*	Initial number of effective rotations
*q*(*t*)	Continuous blade-tip sensing signal generated from blade passing S_1_
*t_k_*	Sampling time interval
*q*(*nt_k_*)	Discrete signal from sampling and quantizing *q*(*t*) at time intervals *t_k_*
*ToA*(*nt_k_*)	BTT signal obtained through ToA extraction algorithm
*ToA*_1_(*nt_k_*)	Initial BTT signal of S_1_
*ToA*_2_(*nt_k_*)	Initial BTT signal of S_2_
*ToA*_1_(*j*,*k*)	Initial BTT signal of S_1_ expressed in terms of each blade and per rotation
*ToA*_2_(*j*,*k*)	Initial BTT signal of S_2_ expressed in terms of each blade and per rotation
*q*_0_(*nt_k_*)	Initial OPR signal of S_0_
*T*_0_(*k*)	Initial OPR signal of S_0_ expressed in terms of per rotation
Δ*ToA*_12_(*j*,*k*)	BTT signal difference between S_1_ and S_2_
Δ*ToA*_12_(*j*)	Average of rotation part of △*ToA*_12_(*j*,*k*)
*T’*	Test duration after angular change
*n_r_’*	Rotational speed after angular change
*K’*	Effective number of rotations after angular change
Δ*ToA*_12_*’*(*j*,*k*)	BTT signal difference between S_1_ and S_2_ afte angular change
Δ*ToA*_12_*’*(*j*)	Average of rotation part of △*ToA*_12_*’*(*j*,*k*) after angular change
*T*_0_’(*k*)	OPR signal of S_0_ expressed in terms of per rotation after angular change
Δ*ToA_j_*	Measured ToA difference before and after S_1_ rotation
Δ	BTT measurement error
*O*	Theoretical center of rotation
*R*	Center of angular change
*S*	Initial position of S_1_
*S’*	Position of S_1_ after angular change
*l_RS_*	Distance between *S* and *R*
*φ*_1_	Eccentricity angle formed by eccentricity error between rotation and angular change
*e*_1_	Eccentricity magnitude formed by eccentricity error between rotation and angular change
*δ*	Non-parallel angle formed by non-parallelism error between rotation and angular change
*l_OS_*	Distance between *S* and *O*
*l_SS’_*	Distance between *S* and *S’*
*l_OS’_*	Distance between *S’* and *O*
*β*	Actual rotation angle of S_1_
*β*-*α*	Angular change-induced eccentricity and non-parallelism error
*O’*	Actual center of rotation
*φ*_2_	Rotation eccentricity angle
*φ*_02_	Initial rotation eccentricity angle
*e*_2_	Rotation eccentricity magnitude
Δ*φ*	Real-time rotation eccentricity angle
*γ_S_*	Measured angle of S_1_ at position *S*
*γ*_0*S*_	Initial measured angle of S_1_ at position *S*
*γ_S_’*	Measured angle of S_1_ at position *S’*
*γ*_0*S*_*’*	Initial measured angle of S_1_ at position *S’*
*γ*	Measured angle of S_1_
Δ*O’SS’*	Virtual triangle formed by angular change and rotation
*l_O_**_’_**_S_*	Distance between *S* and *O’*
*l_O_**_’_**_S’_*	Distance between *S’* and *O’*
*γ*-*β*	Rotation eccentricity error considering angular change-induced eccentricity and non-parallelism
*γ*-*α*	Eccentricity and non-parallelism errors from rotation and angular change
*α*_1_	Initial angle of the multi-tooth indexing table
*α*_2_	Angle of the multi-tooth indexing table after angular change
*θ_E_*	Uncertainty component due to indication error of the multi-tooth indexing table
*θ_ie_*	Uncertainty component due to eccentricity from inhomogeneity of the coaxial positioning device and BTT sensor fixture
*θ_inp_*	Uncertainty due to non-parallelism from inhomogeneity of the coaxial positioning device and BTT sensor fixture
*θ_re_*	Uncertainty component due to rotor rotational eccentricity
*θ_mr_*	Uncertainty component from repeatability of angle measurement of the multi-tooth indexing table
*ω_rsf_*	Uncertainty component from rotor rotational speed fluctuations
*ω_mr_*	Uncertainty component from rotational speed measurement repeatability during rotor rotation
*ε_m_*	Uncertainty component from environmental fluctuations (e.g., temperature, random noise) during testing
Δ*ToA*_12_	ToA difference measurement value between S_1_ and S_2_
*ω_m_*	Rotational speed measurement value
*α_m_*_12_	Angle difference measurement value between S_1_ and S_2_
*α_m_*	Angular change measurement value
*Val*_Δ*ToA*_	Measured value of the BTT measurement system
*Val*_Δ*t*_	Reference value of the BTT accuracy evaluation device
*R*^2^	Coefficient of determination
SD	Standard deviation
RSD	Relative standard deviation
